# Clinical outcomes in patients with neurological disorders following periacetabular tumor removal and endoprosthetic reconstruction of the hemipelvis

**DOI:** 10.3389/fsurg.2024.1279179

**Published:** 2024-03-05

**Authors:** Jichuan Wang, Zhiqing Zhao, Haijie Liang, Jianfang Niu, Xingyu Liu, Han Wang, Yi Yang, Taiqiang Yan, Wei Guo, Xiaodong Tang

**Affiliations:** The Musculoskeletal Tumor Center, Peking University People’s Hospital, Xicheng District, Beijing, China

**Keywords:** neurological conditions, Parkinson’s disease, periacetabular musculoskeletal tumors, hemipelvis reconstruction, clinical outcomes

## Abstract

**Background:**

Surgical treatment of musculoskeletal tumors in the periacetabular region present extremely difficult due to the complex anatomy and need for reconstruction. Orthopedic surgeons face more difficulties in patients with neurological conditions, which can cause increased muscle tone, an elevated risk of fractures, and compromised bone quality. There is limited evidence regarding endoprosthetic reconstruction for periacetabular tumors in individuals with neurological disorders.

**Methods:**

We conducted a single-center retrospective study to examine the outcomes of patients with preexisting neurological conditions who underwent surgery to remove periacetabular tumors and who underwent endoprosthesis reconstruction. Clinical presentation, detailed neurological conditions, complications, and functional outcomes were studied.

**Results:**

Sixteen out of the 838 patients were identified (1.91%), with a mean follow-up time of 33 months. The primary neurological conditions encompassed Parkinson's disease, Alzheimer's disease, dementia, and cerebral ischemic stroke. Every patient was diagnosed with periacetabular lesions that were either primary or oligometastatic. They underwent tumor resection and subsequently received endoprosthetic reconstruction of the hemipelvis. Three patients developed metastasis lesions later, and two patients experienced tumor recurrence. Five cases experienced hip dislocation—one with periprosthetic fracture and one with surgical site infection. The position of the prosthetic rotating center was not correlated with dislocation. The reoperation rate was 31.25%. The cohort of patients all presented with more extended hospital stays and rehabilitation. In 3 patients, the general functional score was good, while in 6 patients, it was fair; in 7 patients, it was regarded as poor. The average MSTS93 score was 49.71%.

**Conclusion:**

Endoprosthetic reconstruction after periacetabular tumor resection is an effective way to eliminate tumors and salvage limbs. However, this group of patients has an increased likelihood of secondary surgery, complications, extended hospital stay, and no significant improvement in functional outcomes. Despite the diverse nature of the cohort, it is recommended to consider enhanced soft tissue reconstruction, supervised functional recovery and rehabilitation training.

## 1 Introduction

Pelvic tumors account for approximately 20% of all primary sarcomas. Surgical management of malignant bone tumors involving the pelvic girdle is often accompanied by significant challenges (1–3); this could be due to significant bone and muscle removal, complex reconstructions, lengthy surgeries, or weakened immune systems after systemic treatment (4–7). In the past 30 years, advancements in surgery have led to several techniques for reconstructing the pelvis, with endoprosthetic reconstruction being a more prominent method than hip transposition or amputation (4, 7–9). Despite a certain incidence of complications, studies conducted globally have indicated that endoprosthetic reconstruction offers a viable solution for preserving limbs and potentially restoring their functionality despite variations in prosthesis design (10–12).

Parkinson's disease (PD) and other neurological disorders cause neuron degeneration, leading to symptoms such as stiffness and tremors. The prevalence of these conditions is increasing substantially, affecting an estimated six million people worldwide (13). Excessive muscle tension around the hip, caused by spasticity, tremors, and contracture, leads to changes in the forces applied to the hip joint (14). Consequently, subluxation, degenerative conditions, and secondary skeletal abnormalities are significantly more prevalent. Patients with neurological disorders who have postural instability are more likely to experience a fall, decreased bone mineral density, physical impairment, or immobilization-induced hypercalcemia (15). Orthopedic surgeons find it challenging to treat these patients, especially during hip replacement, due to the increased risk of dislocation, implant loosening, increased risk of fracture and early prosthesis failure, particularly during hip arthroplasty, which can lead to increased mortality and complications (11, 14, 16–19).

Previously, our center published the most extensive group of patients to date who underwent surgical removal of pelvic tumors and prosthetic reconstruction (4, 20–22). However, for patients with both periacetabular tumors and neurological issues, there is a lack of information on the best care approach during surgery. Here, we aimed to fill this gap by conducting a retrospective study. We wanted to assess the clinical outcomes of patients with preexisting neurological conditions who underwent surgical resection of periacetabular tumors and subsequent prosthetic reconstruction.

## 2 Materials and methods

### 2.1 Patients

The study adhered to the Helsinki Declaration and was approved by the ethics committee at Peking University People's Hospital. We conducted a retrospective, single-center study in which patients diagnosed with resectable periacetabular bone malignancy underwent periacetabular tumor excision and subsequent modular hemipelvic endoprosthesis reconstruction between August 2012 and February 2021 were included. Of the 838 patients that were initially identified, patients who lacked the following neurological conditions or symptoms at admission were excluded: (1) had symptomatic Parkinson's disease, (2) had symptomatic cerebral ischemic stroke, (3) had ataxia, (4) had epilepsy and seizures, and (5) had a history of intracranial surgery with neurological symptoms. Additionally, patients with less than six months of postoperative in-person follow-up were excluded. Ultimately, 16 patients were selected for detailed analysis. Table 1 presents a detailed summary of the demographic and baseline characteristics. Patients diagnosed with primary bone sarcoma received adjuvant chemotherapy, while those with bone metastatic lesions were treated according to established clinical protocols.

**Table 1:** Patient demographics and baseline characteristics.

Cases	Age (years)	Gender	Pathological diagnosis		Neurological conditions/symptoms		Surgical resection type	ASAC	Preop ECOG score
1	68	M	Metastatic	Renal carcinoma	PD	Dystonia, tremor, bradykinesia	II + III	3	3
2	68	F	Primary	Chondrosarcoma	PD	Dystonia, tremor, bradykinesia	II + III	2	3
3	55	F	Primary	Chondrosarcoma	Cerebral ischemic stroke	Dystonia, Hemiplegia	II + III	2	3
4	47	M	Metastatic	hemangiopericytoma	hemangiopericytoma	Dystonia, Bradykinesia	II + III	2	3
5	21	M	Metastatic	Solitary fibrous tumor	Solitary fibrous tumor of skull base	Dystonia, ataxia	I + II	2	3
6	78	M	Primary	Chordoma	PD	Dystonia, tremor, bradykinesia	II	2	3
7	47	F	Primary	Chondrosarcoma	Cerebral ischemic stroke	Dystonia, Bradykinesia	I + II + III	1	3
8	61	M	Metastatic	hemangiopericytoma	Hemangiopericytoma of meninges	Dystonia, flexed posture	II + III	2	3
9	69	M	Primary	Chondrosarcoma	Cerebral ischemic stroke	Dystonia, Bradykinesia, flexed posture	II + III	2	4
10	53	F	Primary	UPS	Ataxia	Dystonia	II	2	3
11	77	M	Metastatic	Renal carcinoma	PD	Dystonia, Tremors, contracture	II	3	4
12	60	F	Primary	Chondrosarcoma	Cerebral ischemic stroke	Dystonia, Tremor, bradykinesia	II + III	2	3
13	50	M	Primary	UPS	Ataxia	Dystonia	I + II + III	2	3
14	60	F	Primary	Chondrosarcoma	Cerebral ischemic stroke	Dystonia, Dementia, Bradykinesia,	I + II	2	3
15	43	M	Primary	Chondrosarcoma	Epilepsy and Seizures	Dystonia, Tremors, contracture	I + II + III	2	4
16	71	M	Primary	Chondrosarcoma	Cerebral ischemic stroke	Dystonia, Bradykinesia,	II	2	3

M, male; F, female; PD, Parkinson's disease; UPS, undifferentiated pleomorphic sarcoma; ASAC, American Society of Anesthesiologists Classification; ECOG, score—Eastern Cooperative Oncology Group Score.

### 2.2 Implant design

**Figure 1:**
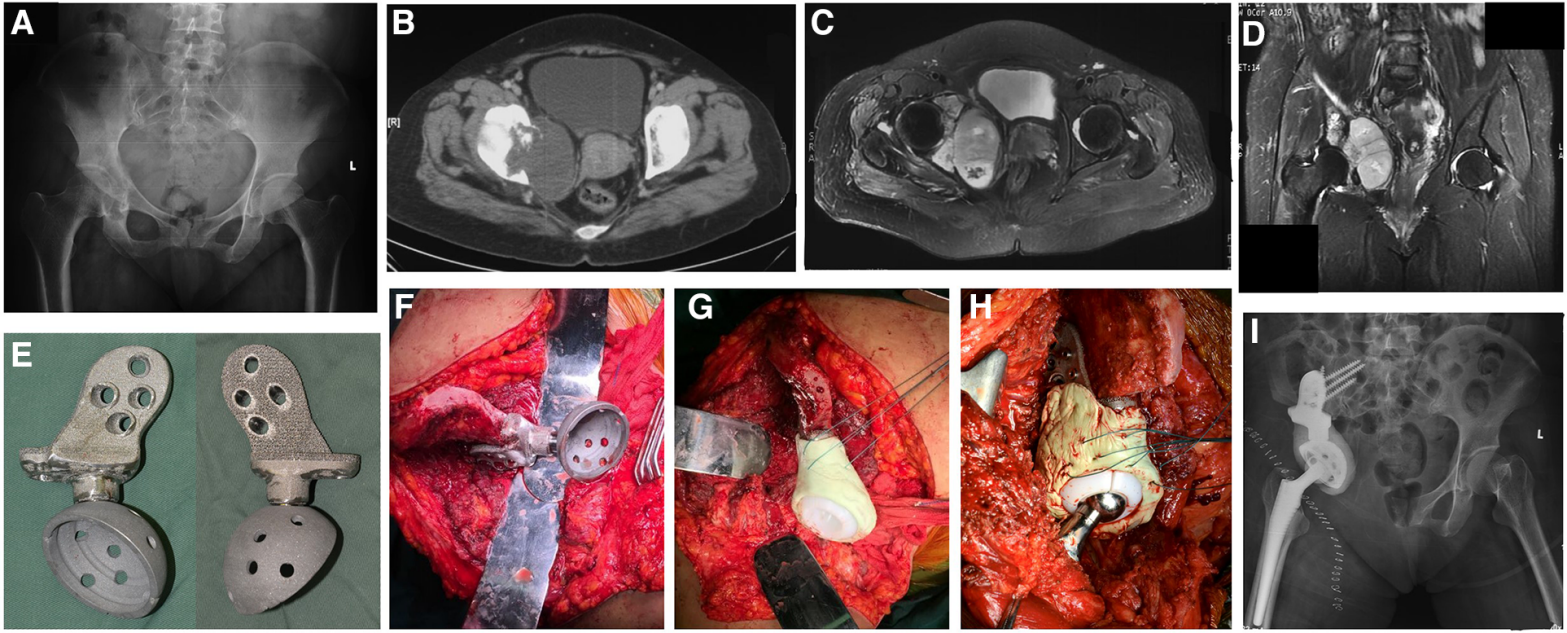
Periacetabular tumor resection and prosthetic reconstruction. Preoperative x-ray (**A**), CT (**B**) and MRI (**C,D**) images showing Zone II + III chondrosarcoma patients with periacetabular involvement. (**E**) Anterior and posterior images showing the design of a hemipelvic prosthesis with a 3D printing interface mimicking trabecular bone. (**F–H**) Intraoperative image showing fixing the hemipelvic prothesis to the remaining ilium, attaching the polyethylene bushing to the prothesis with bone cement, and reducing the hip with a total hip arthroplasty component from the proximal femur. (**I**) Postoperative x-ray.

Modular hemipelvic prostheses were utilized following previously described methods (4, 20, 22). Although the specific design may have evolved over a decade, the fundamental elements of the modular hemipelvis implant utilized in this study encompassed the iliac, acetabular, and femoral components. The iliac components were secured using 2–4 screws on the resected surface of the ilium. The acetabular component, featuring variable heights determined during preoperative planning, was pressure-fitted to the iliac component. Moreover, the femoral component, comprising both the head and stem, also features a press-fit design and is connected to the acetabular component using a polyethylene lining, conforming to the total hip design commonly employed in total hip arthroplasty (THA) (Figure 1).

### 2.3 Surgical technique

The standardized surgical procedures for soft tissue exposure, tumor removal, and prosthetic reconstruction were similar to those outlined in our previously published studies (4, 20, 22). Although the details varied between patients, the principles were the same. We employed a modified ilioinguinal and Smith–Peterson approach. The osteotomies were executed at predetermined levels based on preoperative imaging using a Gigli saw. The involved adjacent structures were partially or totally excised as necessary to achieve wide margins. If muscles were not involved, they were simply detached or left intact, depending on the needs of the exposure. The hip joint capsule was dissected and T-shaped to dislocate the hip joint, after which the patient was kept for subsequent reconstruction. The prosthetic iliac component was attached to the residual ilium. The acetabular components were positioned at 45° inclination and 15° anteversion following anatomical landmarks with fluoroscopy. To minimize the chances of postoperative dislocation, we employed a restricted linear device. Nonabsorbable stitches or synthetic mesh were secured in the periacetabular cement to strengthen the hip joint capsule. Upon finishing the reconstruction, the remaining gluteus was affixed back to either the ilium or the abdominal muscles when the ilium had been resected (Figure 1).

### 2.4 Perioperative management and rehabilitation

In each patient, a multidisciplinary team (MDT) consultation was conducted with the participation of neurology and anesthesia specialists prior to surgery. Medications prescribed for neurological conditions were managed by the neurological team and continued during the perioperative period. Patients with Parkinson's disease (PD) underwent specific evaluations to assess their circulatory and respiratory systems as well as their swallowing function. General anesthesia was administered to all patients, with careful monitoring during extubation and the selection of appropriate antagonists to minimize the risk of seizure induction. Sodium valproate was prophylactically administered to patients with a prior history of epilepsy. Cephalosporin antibiotics were given intravenously during the first week and subsequently administered orally for an additional week after the surgical procedure. The routine use of antithrombotic stockings and anticoagulants was employed. Patients were advised to rest mainly on bed rest for four weeks, during which external hip rotation and flexion were limited to less than 90 degrees using a hip brace. Gradual progression to standing and walking was guided by therapists. The Musculoskeletal Tumor Society 93 (MSTS-93) score was used to assess functional outcome (23).

#### 2.4.1 Clinical and radiographic evaluation

Clinical and radiographic status was assessed at intervals of 3 months during the initial 2-year postoperative period, followed by assessments every 6 months. Follow-up for patients with metastatic disease was coordinated with ongoing surveillance. Pelvic radiographs and CT images taken immediately after surgery and at the latest follow-up were evaluated for positioning, acetabular cup tilt, loosening and shifting of the prosthetic hip rotation center in comparison with those of the contralateral side. The inclination of the acetabular cup was defined as the angle between the border of the cup and the teardrop line (24). Variations greater than 3 degrees of inclination and more than 5 mm in distance between the initial and follow-up radiographs were interpreted as indications of instability (25) (Figure 1).

#### 2.4.2 Statistical analysis

The cumulative complication rate and conditional survival were calculated using the Kaplan‒Meier (K-M) method. Continuous variables are summarized as the means with ranges, whereas categorical variables are recorded as counts with percentages unless stated otherwise. The cumulative probability of remaining free of implant failure was calculated via survival analysis. To compare two variables with discrete outcomes, the chi-square test was employed. The statistical analysis was conducted using IBM SPSS 26.0, with the level of significance defined as *p* < 0.05.

## 3 Results

Our study included 838 participants—437 males and 401 females—who met our criteria. The average age was 41.40 years, ranging from 12 to 81 years. The average duration of follow-up was 33.30 ± 13.18 months, ranging from 14.20 to 68.30 months. Among them, 16 patients were identified for the following study. There were 10 males and six females. The average age was 58.54 ± 14.17 years (range 21–78). Of the 11 individuals who had a primary tumor, 8 (50.00%) were diagnosed with chondrosarcoma, 2 (12.50%) had undifferentiated pleomorphic sarcoma, and 1 (6.25%) had chordoma. Among the 5 patients with a metastatic diagnosis, 2 (12.50%) had hemangiopericytoma, 2 (12.50%) had renal carcinoma, and one (6.25%) had a solitary fibrous tumor. Among the 16 patients with neurological conditions, 4 (25.00%) had PD, 4 (25.00%) had cerebral ischemic stroke, 2 (12.50%) had Alzheimer's disease, and 2 (12.50%) had ataxia; 1 (6.25%) had epilepsy and seizures; and 3 had postintracranial surgery with neurological conditions, 2 of whom had hemangiopericytoma of the meninges, one of whom was a solitary fibrous tumor of the skull base (Table 1). Among the surgical resection types, 7 (43.75%) had type II + III (acetabular, pubic and ischium), 4 (25.00%) had type II (acetabular), 2 (12.50%) had type I + II (ilium and acetabular), and 3 had type I + II + III (hemipelvis) tumor resection and reconstruction (Table 1).

### 3.1 Radiographic evaluation

One acetabular component instability was detected on radiographic evaluation without clinical symptoms after 35.1 months. Breakage of 1 lumbar-prosthetic screw was detected in one patient. No significant prosthesis shift was observed other than these two cases. During the latest follow-up, the assessment of anteroposterior pelvic radiographs revealed an average vertical variation of 2.16 mm (ranging from 0.5 mm to 4.2 mm) between the reconstructed acetabular cup and the opposite acetabulum, with the latter being more cephalic. Moreover, there was no significant difference between the dislocated and non-dislocated groups (*p* = 0.158). The average horizontal distance from the midline to the center of the femoral head was 9.58 cm (range, 7.8 cm–11 cm) on the affected side, while it was 9.78 cm (range, 7.5 cm–12 cm) on the opposite side. This difference was not statistically significant (*p* = 0.26). Moreover, there was no significant difference between the dislocated and non-dislocated groups (*p* = 0.711) (Table 2).

**Table 2:** Oncologic and functional outcomes and complications at the last follow-up.

Cases	Follow-up (months)	Oncologic outcome		Function outcome		Complications	Time to complications	ECOG at last follow-up	Vertical difference(mm)	Horizontal distance (cm)	
		R/M	Patient status	Overall	MSTS93 score (%)					Surgery site	Non-surgery site
1	46	N	AWOD	Poor	45.2	Dislocation	7.6	2	1.2	10	11
2	35	N	AWOD	Poor	35.3	Dislocation, periprosthetic fracture	10.9	3	2	10	10
3	36	N	AWOD	Poor	39.6			2	2.5	9	9.5
4	30	M	AWD	Good	65.8	Dislocation	3.9	2	3	11	11
5	14	M	DOD	Fair	53.2			2	3.5	10.5	10
6	24	N	AWOD	Fair	45.9			2	1	11	11.5
7	34	N	AWOD	Poor	33.5	Surgical site infection	15.3	3	4	10	9
8	14	M	DOD	Fair	55.7			2	2	9	9
9	30	N	AWOD	Fair	57.5			3	1.5	10	11
10	39	N	AWOD	Fair	49.6			2	1	8	8
11	40	N	AWOD	Poor	48.4			3	0.5	10.5	11
12	32	R	AWOD	Good	65.5		23.7	2	1.5	8	8.5
13	21	R	AWD	Poor	39.4	Dislocation, breakage of 1 lumbar-prosthetic screw	21.3	3	4.2	10.5	12
14	68	N	AWOD	Fair	53.8			2	2.1	7.8	7.5
15	35	N	AWOD	Poor	35.9	Dislocation	4.5	3	3.5	10	9
16	25	N	AWOD	Good	61.5			1	1	8	8.5

N, none; M, metastatic lesions; R, recurrence; AWOD, alive without disease; AWD, alive with disease; DOD, died of disease; ECOG, Eastern Cooperative Oncology Group Score.

### 3.2 Implant survival

**Figure 2:**
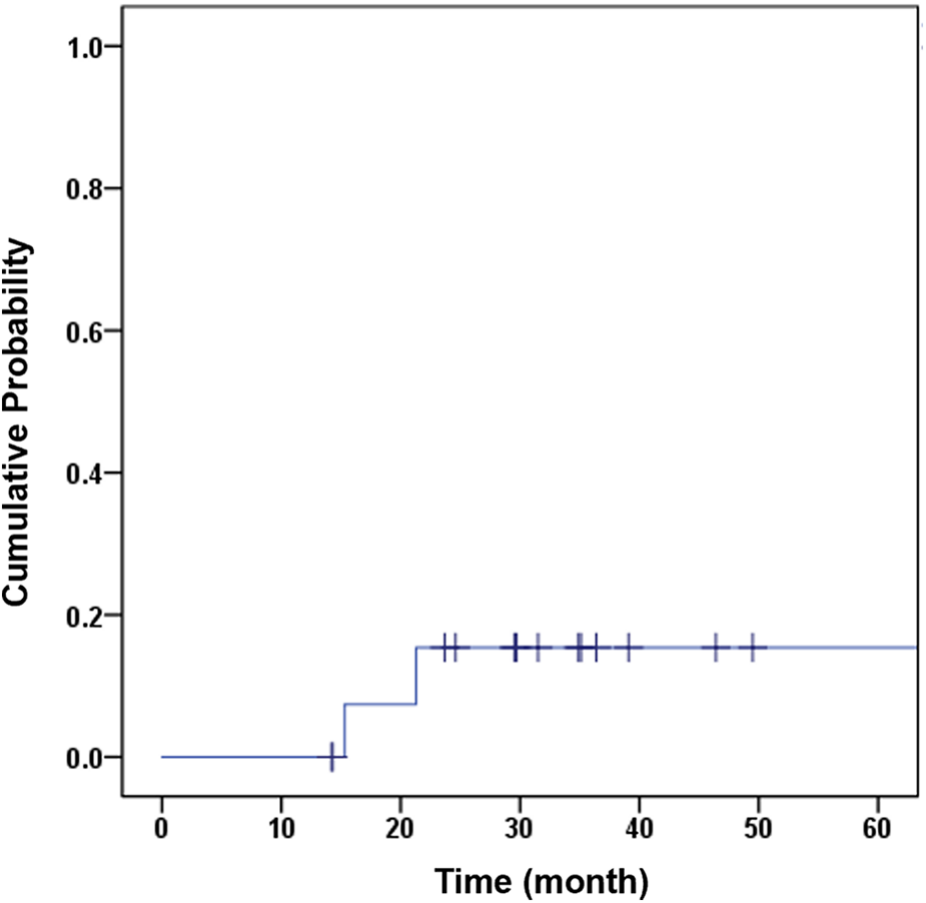
K–M curve with the cumulative probability of implant removal for any reason as the endpoint.

Two patients, accounting for 12.50% of the total patients, necessitated the removal of implants due to either infection (one patient) or local recurrence (one patient). K‒M analysis revealed an 85.7% survival rate for prostheses at the end of 3 years, considering all-cause removal as the endpoint (Figure 2). At a mean duration of 33.3 months, the rate of prosthesis survival was 100% when aseptic loosening was considered the endpoint (Table 2).

### 3.3 Oncological outcome

At the latest follow-up, a total of 11 patients, comprising nine with primary tumors and two with metastatic disease, were alive without any signs of disease recurrence. After surgery, two individuals with metastatic illness succumbed to the disease within an average duration of 14 months. Among the 16 patients, 3 had distant metastasis, which occurred 19 months after the operation on average (with a range of 14–29 months). Two individuals (12.50%) experienced local recurrence after an average of 22 months following surgery. One of these patients underwent radiotherapy for treatment, while the other was managed through limb-sparing surgery (Table 2).

### 3.4 Functional outcome

At the latest follow-up, the average MSTS 93 score was 49.7 (1%), ranging from 33.5% to 68.5%. Patients with various resection types had mean functional scores of 53.50% for Type I + II, 36.27% for Type I + II + III, 53.10% for Type II, and 52.51% for Type II + III. The functional outcome for type I + II + III (hemipelvis) reconstruction was the worst among all four types (*p* = 0.004 vs. type I + II (ilium and acetabular); *p* = 0.044 vs. type II (acetabular); *p* = 0.068 vs. type II + III (acetabular, pubic and ischium)), and the difference was significant. Before surgery, the average Eastern Cooperative Oncology Group (ECOG) score was 3.19 (range, 3–4), while the average score after surgery was 2.31 (range, 1–3) during the final follow-up. All patients achieved no worsening of Eastern Cooperative Oncology Group (ECOG) score after surgery (*p* < 0.005) (Table 2).

### 3.5 Complications

**Figure 3:**
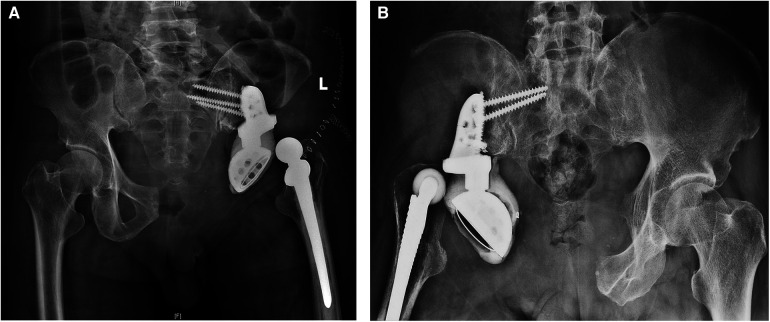
Postoperative complications after prosthetic hemipelvic reconstruction. (**A,B**) Postoperative x-ray showing dislocation of the hip after prosthetic hemipelvic reconstruction in two patients.

A total of 6 complications were recorded in 16 patients (37.5%), all of whom required further surgical intervention (Table 2). Furthermore, two instances of wound dehiscence were addressed through debridement while ensuring no involvement of the deep fascia. The most frequent complication was dislocation, which occurred in 5 patients (31.25%) approximately 9.64 months after surgery (Figure 3). Two cases of dislocation were due to inadvertent falls and caused a periprosthetic fracture in one patient and a breakage of lumbar-prosthetic screws in the other. For the remaining three dislocation cases, closed reduction was attempted under anesthesia with fluoroscopy; one succeeded, and the other two were further treated with open reduction. Deep infection occurred in 1 patient (6.25%) at 15.3 months postsurgery. The patient failed debridement, antibiotics, and implant retention (DAIR) procedures and ultimately underwent implant removal, and methicillin-resistant *Staphylococcus aureus* (MRSA) was found in this patient.

## 4 Discussion

Patients with PD and other neurological disorders frequently experience stiffness, dystonia, adiadochokinesia, difficulty coordinating movements, muscle contractions, and shaking, which are theoretically believed to be accompanied by increased complications and worse mortality after orthopedic surgery (12, 13, 18, 19). PD itself is also a progressive disorder affecting life expectancy. Pelvic malignancies around periacetabular regions often require resection and adjuvant treatment, including radiotherapy or chemotherapy. Surgery usually involves excessive bone and muscle dissection, resection, and complex reconstructions with megaprostheses. To date, hemipelvic prostheses have resulted in relatively high rates of complications (4, 5, 7, 10, 26). Due to the urgent need for surgical treatment, additional risks and adverse expectations have been made for periacetabular tumor patients with underlying neurological conditions, and insults could add to injury. However, due to the low incidence and heterogeneity of this disease in this subgroup of patients, this impression has rarely been proven by clinical studies. Benefiting from our institution's extensive series of cases and consistent surgical techniques for pelvic tumors for several years, we can now report the actual clinical presentation. In this study, we compiled information on the clinical presentation, patient survival, complications, and functional outcomes of individuals with neurological conditions who underwent periacetabular tumor resection and prosthetic reconstruction using hemipelvis megaprostheses. To our knowledge, this is the largest cohort published to date on this topic.

Neurological conditions such as PD are progressive disorders believed to affect life expectancy (27). Several studies have indicated less-than-ideal results and higher perioperative mortality rates in the PD population undergoing THA (14). However, studies have also suggested that PD did not result in an increased risk of death in the early postoperative period after THA. Nevertheless, PD patients experience a greater long-term risk of death (11). Unlike studies conducted on individuals who underwent total hip arthroplasty (THA), musculoskeletal cancer patients with varying life expectancies were included in this study. Compared to our center's historical control of patients without neurological conditions, overall survival was not significantly different (Table 3) (4, 20, 22). Speculating the proportion of risk attributed to cancer progression itself, rather than neurological conditions, plays a more dominant role in overall survival. Overall, in this study, we did not observe a decrease in OS in patients with neurological conditions. However, this finding is also challenging due to the heterogeneity of this patient cohort. Although this approach is expected to be very difficult, further studies with more homogeneous patient cohorts could be more efficient at addressing this topic.

**Table 3:** Studies of hemipelvic endoprosthetic reconstruction after tumor resection in the literature in the past 5 years.

Study	Time	Country	Number of cases	Prosthesis type	Duration of average follow-up (mo)	Functional outcome score (%)	Death rate (%)	Wound dehiscence rate (%)	Deep infection rate (%)	Dislocation rate (%)	Aseptic loosening rate (%)	Implant removal rate (%)
Current study	2023	China	16	M	33	49.71 (33.5–68.5)^a^	12.5	13	6	31	0	6
Guo, et, al (22)	2007	China	28	M	30	62 (30–83)^a^	29	14	14	4	0	4
Ji, et al. (20)	2013	China	100	M	53	57.2 (16.7–86.7)^a^	20	10	6	3	0	4
Ji, et al. (4)	2020	China	80	M	33	84 (43–100)^a^	20	10	6	3	0	4
Wang, et al. (28)	2020	China	13	C	27	73.3 (50–90)^a^	NA	15.4	0	0	0	0
Wang et al. (29)	2018	China	11	C	15.5	64 (43.3–83.3)	NA	9	0	18.2	0	0
Lowe et al. (5)	2019	Britain	52	M	36	54 (10–90)^a^	40	4	12	13	0	4
Issa et al. (7)	2018	France	24	M	49	72 (43–87)^a^	13	17	17	17	8	9
Hipfl et al. (8)	2017	Austria	48	M	79.2	71 (27–93)^a^	40	13	12	15	6	4
Bus et al. (6)	2017	Netherlands	47	M	24	70 (30–93)^a^	32	0	30	21	4	9
Abdel et al. (26)	2017	America	10	M	59	75 (49–92) ^b^	10	10	0	33	0	0
Wang et al. (28)	2020	China	26	C	27	77 (50–90) ^b^	0	16	0	0	0	0
Zoccali et al. (30)	2021	Italy	14	C	42	46.3	0	35.7	16.7	0	0	0

M, modular; C, custom-made.

^a^ MSTS93.

^b^ Harris hip score.

Neurological disorders can also lead to musculoskeletal symptoms and conditions, increasing the likelihood of falls and increasing the risk of dislocation, periprosthetic fracture, and implant failure. In this theory, we thought to include patients with various neurological conditions who share similar symptoms for further analysis when facing a small cohort without additional heterogeneity. A study revealed a greater occurrence of dislocation and periprosthetic fracture in individuals with neurological disorders who underwent THA than in those without (19). Similarly, patients diagnosed with PD who underwent THA were found to have an increased likelihood of requiring revision surgery, particularly within the first year following the initial procedure (18). However, relatively low or no incidences of dislocations were also reported in several large population-based studies, demonstrating the weak correlation between neurological conditions and hip instability (13). Compared to THA, hemipelvis reconstruction results in markedly greater complication rates, regardless of the type of reconstruction performed across institutions (4–8, 20, 22, 26). The massive loss of acetabular bone, periacetabular muscles, ligaments, and hip joint capsules contaminated by the tumor resulted in a lack of stable structures. Hence, a longer operation time, complicated surgical techniques, and additional blood loss also increase the risk of developing postoperative complications. However, since tumor control is dominant in patients with malignancies, orthopedic oncologists are tied to their hands when balancing tumor resection and limb-salvaging strategies. In this scenario, hemipelvis endoprosthesis reconstruction provides opportunities for rebuilding the acetabular cup and reattachment of the remaining muscles and hip joint capsules; this approach is more efficient and superior than other limb-salvaging reconstruction methods, including hip transposition (Table 3) (1, 4, 6, 20). In our study, despite these limitations, orthopedic oncology surgeons and engineers are still attempting to minimize complications and improve postoperative limb function via radical removal of tumors (4).

THA has a lower dislocation rate than hemipelvic endoprosthetic reconstruction (7, 10). Several earlier investigations, including ours, have documented a dislocation rate of approximately one-third following hemipelvic endoprosthetic reconstruction (1, 21, 26, 31). Table 3 displays a comparison of the dislocation rates reported in various studies, including our own. Interestingly, a higher dislocation rate was discovered for the first time in patients with neurological conditions in previous studies. With respect to the factors contributing to dislocation, Wang et al. reported that older age was associated with a greater dislocation rate (10). A similar tendency was reported in patients after THA or proximal femur replacement (32, 33). Elderly individuals often experience decreased muscle strength, reduced bone quality, and an increased likelihood of falling as they age. Neurological conditions such as PD also tend to occur in senior patients. Symptoms such as rigidity, contractures and tremors in senior patients increase the risk of dislocation.

The positioning of the prosthetic acetabular cup tilt and a shift in the prosthetic hip rotation center were also reported as risk factors for dislocation in hemipelvis patients; interestingly, these factors were not found to be significant in this cohort (4). Additionally, the loss of the abductor and iliopsoas after tumor resection disrupted the patient's stability. Despite the lack of correlation between neurological conditions and dislocation tendency in patients who underwent hemipelvis reconstruction in previous studies, we confirmed a significant increase in the dislocation rate within this group of patients (Table 3). Although further research is needed due to the heterogeneity and rareness of prevalence in this cohort, we recommend that additional attention be given to patients with neurological conditions after hemipelvic endoprosthetic reconstruction. Possible measures include more detailed education on fall prevention, more careful postoperative turning over, longer bed rest, and the use of a hip abduction brace after surgery. Preservation and reattachment of the gluteus muscles and the hip joint capsule are worth additional attention during reconstruction. An artificial prosthetic mesh could be used to enhance and reconstruct the joint capsule.

Studies have evaluated pain relief and functional outcomes in PD patients after THA. It is generally anticipated that there will be an enhancement in quality of life and a reduction in pain levels (18, 19). To the best of our knowledge, no research has examined the functional outcome in patients with neurological conditions following hemipelvic reconstruction. As measured by the MSTS93 scoring system, patients with neurological conditions experienced worse functional outcomes after hemipelvis reconstruction than did patients in previously reported studies (Table 3) (5, 7, 20, 22, 26). Furthermore, worse functional outcomes were found in patients who underwent extensive excision and reconstruction (types I + II + III), indicating the importance of the remaining structures after tumor resection for maintaining postoperative function. Additionally, our results showed that hemipelvis reconstruction may not improve quality of life. However, improvements in pain relief were found in the majority of patients. While the surgical goal in this group is different from that of THA for PD patients, which involves removing tumors and reconstructing structures, it is crucial to prioritize promoting early rehabilitation and enhancing functional improvement in the long term. Hence, longer hospitalization times are observed in our cohort and extensive registry studies of PD patients after THA (11). It is essential to prevent surgical complications such as pneumonia, deep vein thrombosis, and urinary tract infection to improve patient quality of life.

This study has several limitations. This study may have inherent selection bias due to its retrospective nature and relatively small sample size. Treatment was not assigned randomly to the patients included in this study. Despite the multidisciplinary consultation for each patient's treatment plan, the surgical team did not rely on standardized protocols for surgical treatment decisions. Moreover, this study included patients with various neurological disorders. Patients also inherit pathological conditions that may be biologically different and affect survival. Despite these limitations, to the best of our knowledge, this study is the first to systematically consider neurological conditions in patients undergoing periacetabular reconstruction. While specific neurological conditions were different, this cohort exhibited similar physical conditions and impacts on the musculoskeletal system, such as abnormal muscle tone, postural instability and low bone quality. As a result, examining clinical manifestations and associated complications in this investigation offers valuable insights and recommendations that can improve clinical practice.

## 5 Conclusion

This study, the largest of its kind, included patients with neurological conditions who underwent periacetabular tumor resection and hemipelvic reconstruction, focusing on survival, complications, and functional outcomes. The findings indicate increased risks of medical complications and prolonged hospital stays without significant functional improvement postsurgery. The need for balanced risk-benefit analysis and patient counseling should be emphasized. This study also suggests enhanced surgical techniques and tailored rehabilitation for better outcomes. Future research should focus on larger, more diverse cohorts to validate these findings and explore various reconstruction techniques and their impact on functional status.

## Data Availability

The raw data supporting the conclusions of this article will be made available by the authors, without undue reservation.

## References

[1] Jaiswal PK Aston WJ Grimer RJ Abudu A Carter S Blunn G Peri-acetabular resection and endoprosthetic reconstruction for tumours of the acetabulum. J Bone Joint Surg Br. (2008) 90:1222–7. 10.1302/0301-620X.90B9.20758 18757964

[2] Falkinstein Y Ahlmann ER Menendez LR. Reconstruction of type II pelvic resection with a new peri-acetabular reconstruction endoprosthesis. J Bone Joint Surg Br. (2008) 90:371–6. 10.1302/0301-620X.90B3.20144 18310763

[3] Khan FA Rose PS Yanagisawa M Lewallen DG Sim FH. Surgical technique: porous tantalum reconstruction for destructive nonprimary periacetabular tumors. Clin Orthop Relat Res. (2012) 470:594–601. 10.1007/s11999-011-2117-2 21989784 PMC3254739

[4] Ji T Yang Y Tang X Liang H Yan T Yang R 3D-printed modular hemipelvic endoprosthetic reconstruction following periacetabular tumor resection: early results of 80 consecutive cases. J Bone Joint Surg Am. (2020) 102:1530–41. 10.2106/JBJS.19.01437 32427766

[5] Lowe M Jeys L Grimer R Parry M. Pelvic reconstruction using pedestal endoprosthesis—experience from Europe. Ann Jt. (2019) 4:34. 10.21037/aoj.2019.06.04

[6] Bus MP Szafranski A Sellevold S Goryn T Jutte PC Bramer JA LUMic(®) endoprosthetic reconstruction after periacetabular tumor resection: short-term results. Clin Orthop Relat Res. (2017) 475:686–95. 10.1007/s11999-016-4805-4 27020434 PMC5289170

[7] Issa SP Biau D Babinet A Dumaine V Le Hanneur M Anract P. Pelvic reconstructions following peri-acetabular bone tumour resections using a cementless ice-cream cone prosthesis with dual mobility cup. Int Orthop. (2018) 42:1987–97. 10.1007/s00264-018-3785-2 29460155

[8] Hipfl C Stihsen C Puchner SE Kaider A Dominkus M Funovics PT Pelvic reconstruction following resection of malignant bone tumours using a stemmed acetabular pedestal cup. Bone Joint J. (2017) 99-b:841–8. 10.1302/0301-620X.99B6.BJJ-2016-0944.R1 28566407

[9] Hu X Lu M Zhang Y Li Z Wang J Wang Y Pelvic-girdle reconstruction with three-dimensional-printed endoprostheses after limb-salvage surgery for pelvic sarcomas: current landscape. Br J Surg. (2023) 110:1712–22. 10.1093/bjs/znad310 37824784 PMC10638540

[10] Wang H Tang X Ji T Yan T Yang R Guo W. Risk factors for early dislocation of the hip after periacetabular tumour resection and endoprosthetic reconstruction of the hemipelvis. Bone Joint J. (2021) 103-b:382–90. 10.1302/0301-620X.103B2.BJJ-2020-0928.R1 33517736

[11] Jämsen E Puolakka T Peltola M Eskelinen A Lehto MU. Surgical outcomes of primary hip and knee replacements in patients with Parkinson’s disease: a nationwide registry-based case-controlled study. Bone Joint J. (2014) 96-b:486–91. 10.1302/0301-620X.96B4.33422 24692615

[12] Fontalis A Kenanidis E Bennett-Brown K Tsiridis E. Clinical outcomes in elective total hip arthroplasty in Parkinson’s disease: a systematic review of the literature. EFORT Open Rev. (2020) 5:856–65. 10.1302/2058-5241.5.200034 33425374 PMC7784138

[13] Newman JM Sodhi N Dalton SE Khlopas A Newman RP Higuera CA Does Parkinson disease increase the risk of perioperative complications after total hip arthroplasty? A nationwide database study. J Arthroplasty. (2018) 33:S162–s166. 10.1016/j.arth.2018.01.006 29402715

[14] Queally JM Abdulkarim A Mulhall KJ. Total hip replacement in patients with neurological conditions. J Bone Joint Surg Br. (2009) 91:1267–73. 10.1302/0301-620X.91B10.22934 19794158

[15] Di Monaco M Vallero F Di Monaco R Tappero R Cavanna A. Bone mineral density in hip-fracture patients with Parkinson’s disease: a case-control study. Arch Phys Med Rehabil. (2006) 87:1459–62. 10.1016/j.apmr.2006.07.265 17084120

[16] Fink HA Kuskowski MA Orwoll ES Cauley JA Ensrud KE. Association between Parkinson’s disease and low bone density and falls in older men: the osteoporotic fractures in men study. J Am Geriatr Soc. (2005) 53:1559–64. 10.1111/j.1532-5415.2005.53464.x 16137287

[17] Meek RM Allan DB McPhillips G Kerr L Howie CR. Epidemiology of dislocation after total hip arthroplasty. Clin Orthop Relat Res. (2006) 447:9–18. 10.1097/01.blo.0000218754.12311.4a 16672897

[18] Wojtowicz AL Mohaddes M Odin D Bülow E Nemes S Cnudde P. Is Parkinson’s disease associated with increased mortality, poorer outcomes scores, and revision risk after THA? Findings from the Swedish hip arthroplasty register. Clin Orthop Relat Res. (2019) 477:1347–55. 10.1097/CORR.0000000000000679 31136433 PMC6554142

[19] Rondon AJ Tan TL Schlitt PK Greenky MR Phillips JL Purtill JJ. Total joint arthroplasty in patients with Parkinson’s disease: survivorship, outcomes, and reasons for failure. J Arthroplasty. (2018) 33:1028–32. 10.1016/j.arth.2017.11.017 29199060

[20] Ji T Guo W Yang RL Tang XD Wang YF. Modular hemipelvic endoprosthesis reconstruction–experience in 100 patients with mid-term follow-up results. Eur J Surg Oncol. (2013) 39:53–60. 10.1016/j.ejso.2012.10.002 23131428

[21] Guo W Li D Tang X Ji T. Surgical treatment of pelvic chondrosarcoma involving periacetabulum. J Surg Oncol. (2010) 101:160–5. 10.1002/jso.21442 19960486

[22] Guo W Li D Tang X Yang Y Ji T. Reconstruction with modular hemipelvic prostheses for periacetabular tumor. Clin Orthop Relat Res. (2007) 461:180–8. 10.1097/BLO.0b013e31806165d5 17452921

[23] Enneking WF Dunham W Gebhardt MC Malawar M Pritchard DJ. A system for the functional evaluation of reconstructive procedures after surgical treatment of tumors of the musculoskeletal system. Clin Orthop Relat Res. (1993) (286):241–6. 10.1097/00003086-199301000-00035 8425352

[24] Massin P Schmidt L Engh CA. Evaluation of cementless acetabular component migration. An experimental study. J Arthroplasty. (1989) 4:245–51. 10.1016/S0883-5403(89)80020-8 2795031

[25] Flecher X Appy B Parratte S Ollivier M Argenson JN. Use of porous tantalum components in paprosky two and three acetabular revision. A minimum five-year follow-up of fifty one hips. Int Orthop. (2017) 41:911–6. 10.1007/s00264-016-3312-2 27766385

[26] Abdel MP von Roth P Perry KI Rose PS Lewallen DG Sim FH. Early results of acetabular reconstruction after wide periacetabular oncologic resection. J Bone Joint Surg Am. (2017) 99:e9. 10.2106/JBJS.16.00803 28145959

[27] Karadsheh MS Rodriguez EK Harris MB Zurakowski D Lucas R Weaver MJ. Mortality and revision surgery are increased in patients with Parkinson’s disease and fractures of the femoral neck. Clin Orthop Relat Res. (2015) 473:3272–9. 10.1007/s11999-015-4262-5 25800376 PMC4562940

[28] Wang J Min L Lu M Zhang Y Wang Y Luo Y What are the complications of three-dimensionally printed, custom-made, integrative hemipelvic endoprostheses in patients with primary malignancies involving the acetabulum, and what is the function of these patients? Clin Orthop Relat Res. (2020) 478:2487–501. 10.1097/CORR.0000000000001297 32420722 PMC7594920

[29] Wang B Hao Y Pu F Jiang W Shao Z. Computer-aided designed, three dimensional-printed hemipelvic prosthesis for peri-acetabular malignant bone tumour. Int Orthop. (2018) 42:687–94. 10.1007/s00264-017-3645-5 28956108

[30] Zoccali C Baldi J Attala D Scotto di Uccio A Cannavò L Scotto G 3D-Printed Titanium custom-made prostheses in reconstruction after pelvic tumor resection: indications and results in a series of 14 patients at 42 months of average follow-up. J Clin Med. (2021) 10(16):3539. 10.3390/jcm10163539 34441834 PMC8397106

[31] Fisher NE Patton JT Grimer RJ Porter D Jeys L Tillman RM Ice-cream cone reconstruction of the pelvis: a new type of pelvic replacement: early results. J Bone Joint Surg Br. (2011) 93:684–8. 10.1302/0301-620X.93B5.25608 21511936

[32] Rowan FE Benjamin B Pietrak JR Haddad FS. Prevention of dislocation after total hip arthroplasty. J Arthroplasty. (2018) 33:1316–24. 10.1016/j.arth.2018.01.047 29525344

[33] Henderson ER Keeney BJ Pala E Funovics PT Eward WC Groundland JS The stability of the hip after the use of a proximal femoral endoprosthesis for oncological indications: analysis of variables relating to the patient and the surgical technique. Bone Joint J. (2017) 99-b:531–7. 10.1302/0301-620X.99B4.BJJ-2016-0960.R1 28385944

